# High-Performance Liquid Chromatographic Separation of Stereoisomers of *ß*-Methyl-Substituted Unusual Amino Acids Utilizing Ion Exchangers Based on *Cinchona* Alkaloids

**DOI:** 10.3390/ijms26094004

**Published:** 2025-04-23

**Authors:** Gábor Németi, Róbert Berkecz, Dániel Ozsvár, Zsolt Szakonyi, Wolfgang Lindner, Aleksandra Misicka, Dagmara Tymecka, Géza Tóth, Antal Péter, István Ilisz

**Affiliations:** 1 Institute of Pharmaceutical Analysis, University of Szeged, Somogyi u. 4, H-6720 Szeged, Hungaryberkecz.robert@szte.hu (R.B.); apeter@chem.u-szeged.hu (A.P.); 2 Institute of Pharmaceutical Chemistry, University of Szeged, Eötvös u. 6, H-6720 Szeged, Hungary; szakonyi.zsolt@szte.hu; 3 Department of Analytical Chemistry, University of Vienna, Währinger Strasse 38, 1090 Vienna, Austria; wolfgang.lindner@univie.ac.at; 4 Faculty of Chemistry, University of Warsaw, Pasteura Str. 1, 02-093 Warsaw, Poland; misicka@chem.uw.edu.pl (A.M.); dulok@chem.uw.edu.pl (D.T.); 5 Institute of Biochemistry, Biological Research Centre, Temesvári krt. 62, H-6725 Szeged, Hungary; toth.geza@brc.mta.hu

**Keywords:** high-performance liquid chromatography (HPLC), enantioselective separation, *ß*-methyl amino acids, *Cinchona* alkaloid-based chiral stationary phases

## Abstract

Novel peptides based on common amino acid building blocks may serve as possible drug candidates; however, their flexible structures may require stabilization via the incorporation of conformational constraints. The insertion of unusual amino acids is a feasible option that may provide improved pharmacokinetic and pharmacodynamic properties of such peptide-type drugs. The stereochemical purity of these kinds of building blocks must be verified by an efficient separation technique, such as high-performance liquid chromatography. Here, we present and discuss the results of the stereoselective separation mechanism of *ß*-methylated phenylalanine (*ß*-MePhe), tyrosine (*ß*-MeTyr), 1,2,3,4-tetrahydroisoquinoline-3-carboxylic acid (*ß*-MeTic), and cyclohexylalanine (*ß*-MeCha) together with non-methylated Phe, Tyr, Tic, and Cha applying *Cinchona* alkaloid-based chiral stationary phases (CSPs). The studied zwitterionic CSPs acting as ion exchangers provided optimal performance in the polar ionic mode when methanol or a mixture of methanol and acetonitrile was utilized as the mobile phase together with organic acid and base additives. It was found that the basicity of small amines applied as mobile phase additives did not directly influence the chromatographic ion exchange concept. However, the size of the amines and their concentration led to a reduced retention time following the principles of ion exchange chromatography. On the basis of a systematic study of the effects of the eluent composition on the chromatographic behavior, important structure–retention and enantioselectivity relationships could be revealed. Through a temperature study, it has become evident that the composition of the eluent and the structure of analytes markedly affect the thermodynamic properties.

## 1. Introduction

The discovery of new drugs with a desired pharmaceutical efficiency is essential for future applications. The design and synthesis of novel molecular structures and the in vitro testing of drug candidates are vital steps in the development process. The conventional way of small-molecule drug discovery is a rather time- and money-demanding procedure. However, automation and the application of the artificial intelligence tools can lead to substantial improvements in the rate and effectiveness of drug discovery [1,2]. Drug design has long been focused on small molecules; however, larger molecules such as peptides may offer improved selectivities due to their specific binding properties.

From a therapeutic point of view, naturally occurring peptides often suffer from poor chemical and physical stability. These deficiencies, however, may be circumvented by dedicated chemical modifications [3]. Since most peptide chains are highly flexible and possess several possible conformations, a feasible approach to stabilize the structure is the insertion of conformational constraints in the peptide chains [4,5]. Unusual (or unnatural, that is, nonproteogenic) amino acids have long been applied as building blocks in organic and medicinal chemistry [6,7]. Therefore, the application of unusual amino acids greatly amplifies the field of peptide design and protein engineering, leading to improved pharmacokinetic properties, enhanced receptor selectivity, and modulated efficacy [8,9]. Several unusual α-amino acid analogs have been designed to restrict side chain functionality, with *ß*-methyl substitution representing a key structural modification employed for this purpose [10]. These new *ß*-methylated *α*-amino acids can be produced synthetically, where their synthesis leads to a mixture of stereoisomers or enantiomer-enriched products, usually far from 100% enantiomeric purity. To control stereoisomeric purity, developing analytical methods for efficient chiral separations and qualitative and quantitative determinations of the *ß*-methyl amino acids and peptides containing them is essential. The procedures used for chromatographic enantioseparations can generally be classified as either indirect or direct methods. Indirect methods have several advantages. These include (i) the derivatization reagents available in a variety of structures; (ii) the separation of the formed diastereomers can be performed on conventional high-performance liquid chromatography (HPLC) columns; (iii) the chromatographic properties of the resulting compound can be influenced during the derivatization process, or the detection limit can be reduced; and (iv) the elution sequence can be inferred (not necessarily requiring the determination of the absolute configuration) and influenced (using an antipode reagent). However, (i) derivatization is a time-consuming task and cannot always be automated; (ii) enantiomers must have a suitable functional group; (iii) the reaction used must be relatively fast and quantitative, (iv) derivatization can cause racemization and/or kinetic resolution; and (v) excess reactant and possible by-products may cause interfering peaks. Since the 1990s, enantioselective separations based on the use of chiral stationary phases have increasingly eclipsed indirect determinations. The main reasons are their favorable properties. Namely, (i) no sample preparation is required; (ii) the chiral purity of the selector is not critical; (iii) enantiomers without a functional group of appropriate reactivity can be separated; (iv) the probability of racemization is negligible; (v) the molar absorbance of the enantiomers is the same, thus making the quantitative analysis easier; and (vi) the individual enantiomers can be recovered after separation, that is, the method is also highly suitable for preparative purposes. However, (i) the formation of stereoselective interactions is not fully understood and the elution order cannot be predicted; (ii) the theoretical plate number is usually smaller; and (iii) there is no universally applicable stationary phase. Due to the advantages mentioned above, direct methods based on the use of HPLC and chiral stationary phases (CSPs) have become dominant in recent years. Theoretically, any chiral molecule is capable of serving as a stationary phase selector (SO), with a diversity of options having been documented. However, chiral selectors (SOs) are typically derived from a limited variety of compound classes. The predominantly utilized chiral SOs include derivatized polysaccharides (such as cellulose and amylose derivatives), cyclodextrins, proteins, as well as macrocyclic compounds like antibiotics and chiral crown ethers. Extensive reviews have thoroughly examined and discussed chiral selectors employed as CSPs [11,12,13,14,15,16,17,18,19,20,21,22,23,24,25].

**Figure 1:**
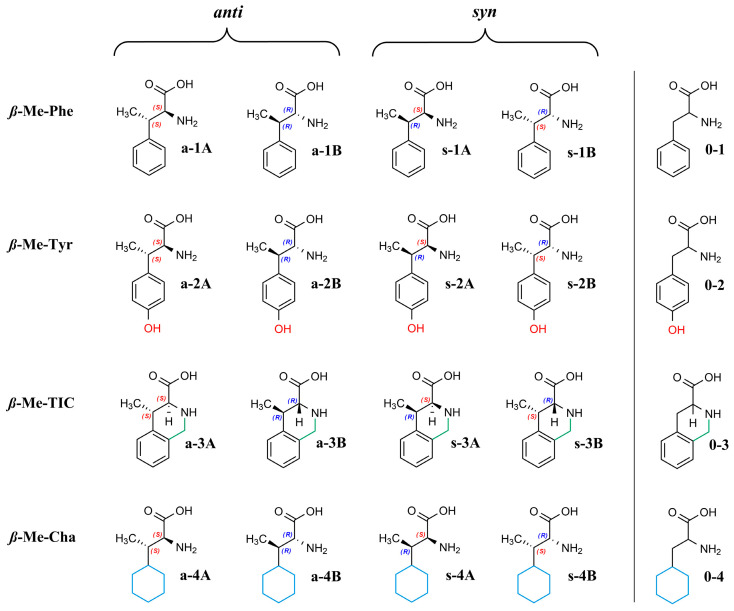
Structures of the analytes.

Due to *ß*-methyl substitution, the α-amino acids studied here possess two stereogenic centers and, therefore, four stereoisomers exist, as illustrated in Figure 1.

For the direct separation of the stereoisomers of *ß*-methyl-phenylalanine (*ß*-MePhe), *ß*-methyl-tyrosine (*ß*-MeTyr), and 4-methyl-1,2,3,4-tetrahydroisoquinoline-3-carboxylic acid (*ß*-MeTic), macrocyclic glycopeptide-based selectors were applied earlier [26,27,28].

Among the various CSPs described in the literature, chiral ion exchangers are characterized as charged SOs, which may provide different stereoselectivities for various types of ionizable chiral analytes. CSPs derived from *Cinchona* alkaloids offer unique selectivity due to their rigid chiral framework and multiple interaction sites. Consequently, they are commonly employed either as weak anion exchangers for the enantioseparation of a variety of chiral acids or as zwitterions for the resolution of anionic, cationic, and ampholytic compounds [18,20]. Since *Cinchona* alkaloid-based CSPs have already shown their suitability in amino acid analysis [20,29,30,31,32,33,34,35], we decided to study and expand their applicability for the stereoisomeric separation of *ß*-methyl-substituted amino acids. In an earlier investigation, a quinine-based chiral anion exchanger was applied for the enantioseparation of *ß*-MePhe, *ß*-MeTyr, and *ß*-MeTic in their derivatized forms [36]. However, this procedure is rather time-consuming and disadvantageous, since any manipulation of analytes before analysis involves the risk of changing the integrity of the analyte.

Here, we present and discuss the results of the investigation of the separation mechanisms of *ß*-MePhe, *ß*-MeTyr, *ß*-MeTic, and *ß*-methylcyclohexylalanine (*ß*-MeCha) together with the non-*ß*-methylated Phe, Tyr, Tic, and Cha in the free form on *Cinchona* alkaloid-based CSPs. To the best of our knowledge, direct separation of *ß*-MeCha is described for the first time in the literature. The study has a number of objectives, namely, (i) to compare and characterize isobaric structurally similar but stereoisomerically different *Cinchona* alkaloid-based CSPs; (ii) to provide some general information on the stereoselective separation mechanism; (iii) to discover possible relationships between thermodynamic data and structural characteristics of the analytes; and, finally, (iv) to develop high-performance liquid chromatography (HPLC)-based methods for the separation of the four stereoisomers of such unusual amino acids. This information may further be utilized in the development of HPLC-based separations of the stereoisomers of various other unusual amino acids.

## 2. Results and Discussion

### 2.1. Stationary Phase Selection

All chiral analytes investigated in this study possess an amino and a carboxylic group in an underivatized form and can be characterized as ampholytes. Under the chromatographic conditions applied, the analytes are expected to be present in their zwitterionic form. Consequently, they can be retained either on a cation or an anion exchanger, but also on a zwitterion-type CSP. Since all CSPs studied are used mainly in the polar ionic mode (PIM) with eluents based on acetonitrile (MeCN) and methanol (MeOH) and acid and base additives [33], a mobile phase containing 50 mM formic acid (FA) and 25 mM triethylamine (TEA) was applied for the screening study. For the screening experiments, first, different single-ion exchangers were probed. No significant enantiomer separations could be achieved with chiral cation exchangers (DML-*RR* and DCL-*SS*) for any of the analytes, while some partial separation could be obtained with the anion exchanger columns (Chiralpak QN-AX and QD-AX). Because variations in the eluent composition did not result in marked improvements in the enantioselective resolutions, to provide information on the stereoselective separation mechanism, all further experiments were carried out with the zwitterionic CSPs with the aim of acquiring information on the stereoselective separation mechanism.

### 2.2. Effect of the Nature of the Applied Additives on the Separation Obtained on Zwitterionic CSPs

**Figure 2:**
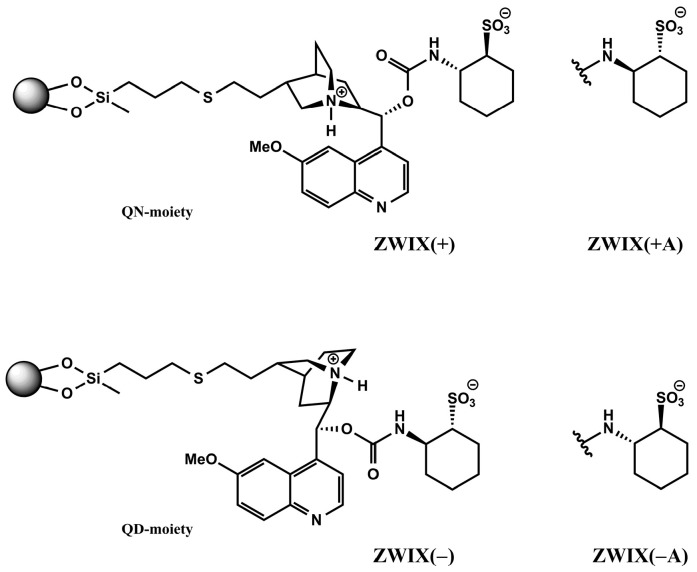
Structures of the employed chiral stationary phases.

*Cinchona* alkaloid-based zwitterionic ion exchanger-type CSPs are designed to interact more or less simultaneously with both the amphoteric analytes and the counterions. By fusing the two subunits—quinine (QN) or quinidin (QD) and (*R,R*)- or (*S,S*)-aminocyclohexanesulfonic acid (ACHSA)—four types of SO can be formed, as illustrated in Figure 2. When these zwitterionic CSPs are applied, the formation of two distinguishable ion pairs is possible at the same time through electrostatic interactions between the weak anion exchanger (tertiary amine) and the strong cation exchanger (sulfonic acid) sites of the SO with the zwitterionic analyte to be separated (e.g., carboxyl and amino groups in the case of amino acids). Secondary interactions such as H-bonding, van der Waals, π–π, and steric interactions will subsequently play important roles in chiral recognition.

**Figure 3:**
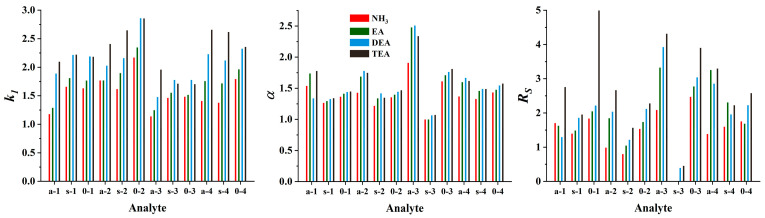
Effects of the nature of the base applied as the mobile phase additive on the chromatographic performance. Chromatographic conditions: column, Chiralpak ZWIX(−); mobile phase, MeOH/MeCN (50:50, *v*/*v*); additives, 50 mM FA and 25 mM base (NH_3_, EA, DEA, TEA); flow rate, 0.6 mL min^−1^; detection, 254 nm, and CAD; temperature, 25 °C; analytes, 1—*ß*-Me-Phe, 2—*ß*-Me-Tyr, 3—*ß*-Me-Tic, 4—*ß*-Me-Cha.

*Cinchona* alkaloid-based CSPs show optimal performance in PIM when a combination of a protic solvent, such as MeOH, and an aprotic solvent, such as MeCN, is used [27]. To facilitate ionic interactions and regulate their effects, including peak shapes, the addition of acid and base additives (acting as counter ions) in the mobile phase is essential. To ensure a protonated form for the quinuclidine group of SOs, an excess of acid is necessary in the mobile phase. Moreover, the acid excess enables a positive charge (through protonation) of both the base additive and the amino group of the analytes. Based on our previous experience [33], a formic acid concentration of 50 mM and a base concentration of 25 mM were chosen with a constant MeOH/MeCN ratio (50:50, *v*/*v*). Using the ZWIX(−) column, ethylamine (EA), diethylamine (DEA), TEA, and NH_3_ (in methanolic solution) were tested as additives to investigate the effect of the nature of the base on the chromatographic characteristics. The protonated amine additive can form a strong ionic interaction with the anionic interaction site of the selector (SO). In other words, they will act as counterions in ion exchange mode; thus, a significant effect on the retention behavior of the charged ampholytic analytes can be expected. As shown in Figure 3, retention factors increased in most cases with increasing substitution of the nitrogen atom.

In several cases, similar trends can be observed with respect to selectivities and resolutions, with *α* and *R_s_* increasing with increasing substitution of the nitrogen atom. Since there is no marked difference between the *pK_a_* values for the organic amines examined (the *pK_a_* values are 10.70, 10.84, and 10.75, for EA, DEA, and TEA, respectively, while it is 9.25 for NH_3_ [37]), it can be stated that the basicity of the amines cannot be directly related to their effects on the chromatographic properties. Rather, structural characteristics are the determining factors. In most cases, the elution strength was TEA ≤ DEA < EA ≤ NH_3_; the larger the counterion size, the weaker its ability to replace the analyte that interacts with the acidic site of the zwitterionic CSP.

In another set of experiments, the effect of acetic acid (AcOH, 50 mM) was also compared with that of FA (50 mM) in the presence of TEA (25 mM) using a ZWIX(−) column. Although retention showed a slight decrease, the resolution was markedly reduced, while selectivity did not change significantly (Table S1). Since FA and TEA provided the best selectivities and resolutions in most cases, these conditions were kept constant in all further experiments.

### 2.3. Stoichiometric Displacement Model for the Characterization of Ionic Interactions

Ion exchange-based CSPs rely on ionic interactions between the analyte and the stationary phase. Additives, such as counterions, function as competitors for ionic interaction sites. The SOs of zwitterionic CSPs contain both anion and cation exchanger functional groups, and thus both anions and cations can be regarded as counterions, enabling an adjustment of retention by altering their concentration. Under these conditions, retention behavior can be explained using the stoichiometric displacement model [38,39,40]. According to this model, there is a direct relationship between the logarithm of the retention factor (*k*) and the logarithm of the counterion concentration (*c_counterion_*). By plotting log*k* against log*c_counterion_* (described in Equation (1)), the slope of the resulting line reveals the effective charge (*Z*), while the intercept describes the ion exchange equilibrium (*K_Z_*).

(1) $$\log\;k=\log\;K_{Z}-Z\;\log\;c_{counterion}$$

**Figure 4:**
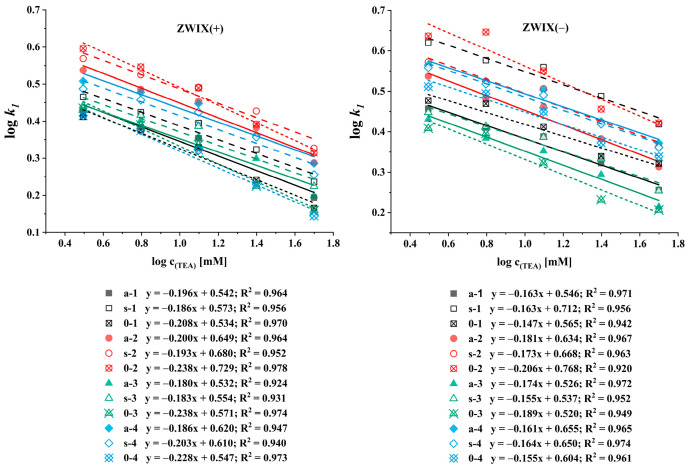
Effects of the counterion concentration on the retention factor of the first-eluting enantiomer. Chromatographic conditions: columns, Chiralpak ZWIX(+), ZWIX(−); mobile phase, MeOH/MeCN (50:50, *v*/*v*); additives, FA (6.25–100 mM) and TEA (3.125–50 mM); flow rate, 0.6 mL min^−1^; detection, 254 nm, and CAD; temperature, 25 °C.

To characterize the retention mechanism observed on the ZWIX(−) and ZWIX(+) columns, the counterion concentration was varied in the MeOH/MeCN (50:50, *v*/*v*) eluents containing FA (ranging from 6.25 to 200 mM) and TEA (ranging from 3.125 to 100 mM) applied in a constant 2:1 ratio. For the quantitative evaluation, the logarithm of the retention factors of the first-eluting enantiomers was plotted against the logarithm of the counterion concentration, as shown in Figure 4. The calculated slopes varied in a rather narrow range from −0.15 to −0.18 for *ß*-methylated amino acids and −0.14 to −0.21 for unmethylated amino acids on the ZWIX(−) column, and from −0.18 to −0.20 for methylated amino acids and −0.21 to −0.24 for unmethylated amino acids on the ZWIX(+) column (Figure 4). For the different *ß*-methylated and unmethylated amino acids, neither the slope nor the intercept differed significantly. These results highlight the effect of the counterion concentration on the chromatographic performance. Although the selectivity and resolution remained largely unchanged, retention decreased with increasing counterion concentrations, consistent with the ion exchange behavior. Compared to the single-ion-type ion exchangers [41], the zwitterionic CSPs are less influenced by the counterion concentration and can be expected to offer better enantiorecognition ability in the analysis of zwitterionic compounds due to the double ion-pairing retention mechanism.

### 2.4. Effects of the Eluent Composition and Structural Properties on the Separation Characteristics

In addition to the effects of the additives mentioned in Section 2.2, the composition of the eluent mixture has a significant influence on the chromatographic behavior of the components to be separated, as they affect the solvation conditions, the ionization of the SO, the counterions, and the compounds to be separated. Gaining knowledge of the effects of eluent composition is essential for a more comprehensive understanding of retention behavior.

Keeping the additive concentration constant (50 mM FA and 25 mM TEA), the MeOH/MeCN ratio was systematically varied within the range of 100/0 to 20/80 (*v*/*v*), enabling us to determine the optimal mobile phase composition and screen interactions between the SO and the analytes. As a result of the reduced solvation effectiveness of polar compounds at higher MeCN concentrations, stronger electrostatic interactions are anticipated. On the other hand, MeOH weakens H-bonding interactions by competing with the analytes for the binding sites of the SO [42].

**Figure 5:**
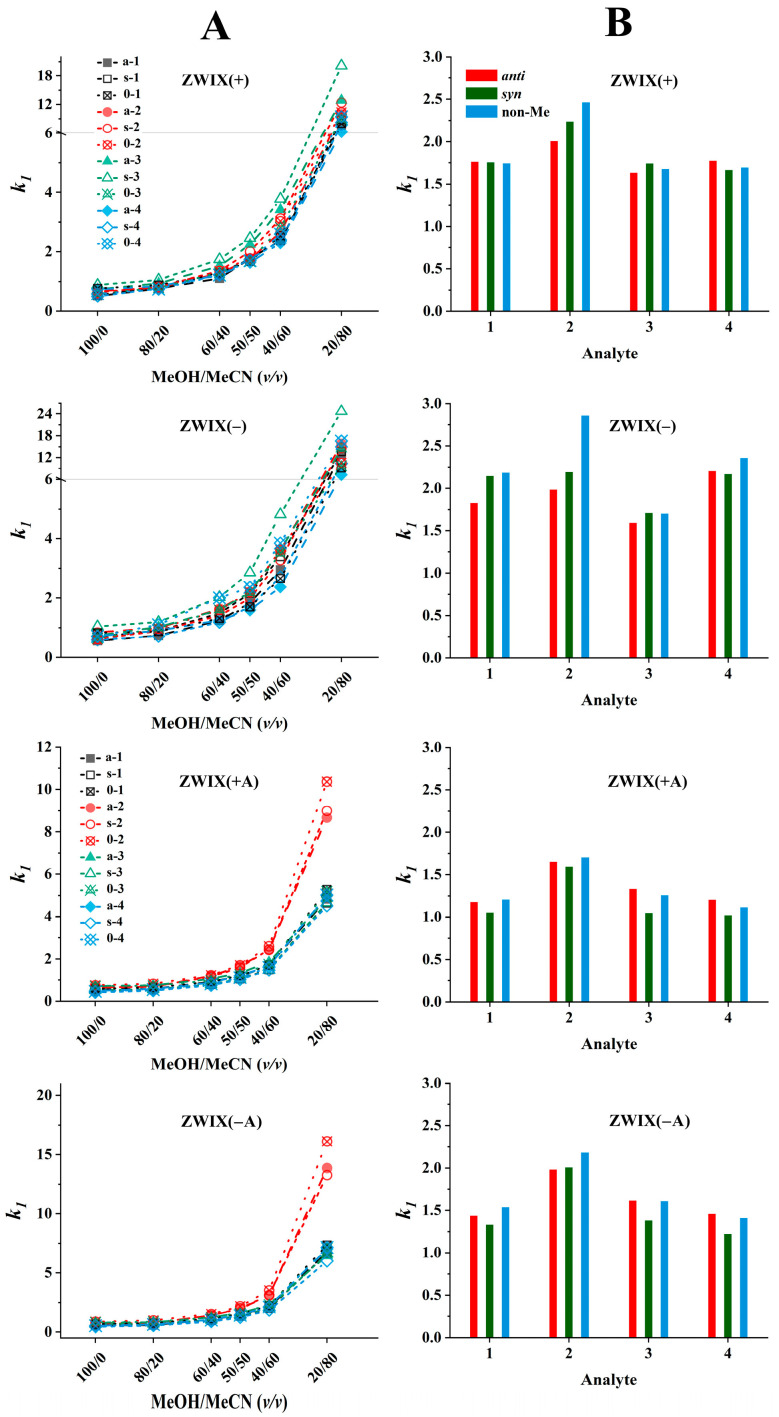
Effects of the mobile phase composition on retention. Chromatographic conditions: columns, Chiralpak ZWIX(+), ZWIX(−), ZWIX(+A), ZWIX(−A); mobile phase, (**A**) MeOH/MeCN (100:0–20:80, *v*/*v*), (**B**) MeOH/MeCN (50:50, *v*/*v*); additives, 50 mM FA + 25 mM TEA; flow rate, 0.6 mL min^−1^; detection, 254 nm, and CAD; temperature, 25 °C; analytes, 1—*ß*-Me-Phe, 2—*ß*-Me-Tyr, 3—*ß*-Me-Tic, 4—*ß*-Me-Cha.

The effects of the bulk solvent composition recorded on the four zwitterionic columns are illustrated in Figure 5A. The peaks eluting first for all analytes increased with higher MeCN contents on all four columns. Relatively small retention factors were measured in MeOH (100%, *v*), while when increasing the MeCN ratio up to 50% (*v*/*v*), a moderate increase, and over 50% (*v*/*v*), an intense increase in retention factors were recorded. The retention patterns observed for each column are quite similar; however, some important differences are worth highlighting. To simplify the presentation, in Figure 5B, the values of *k_1_* measured in the MeOH/MeCN (50:50, *v*/*v*) eluents containing FA (50 mM) and TEA (25 mM) are illustrated. The retention factors of the first eluting peaks varied between 1.5 and 2.7 on the ZWIX(+) and ZWIX(−) columns, and somewhat lower *k_1_* values (1.0–2.0) were recorded with the ZWIX(+A) and ZWIX(−A) columns.

The highest retention was observed for the Tyr analogs on all four columns; probably, the additional OH group on the aromatic ring leads to different H-bonding and/or steric interactions. The retention behavior of the other three compounds on each column was very similar; neither the ring closure (Tic analogs) nor the absence of the aromatic system (Cha analogs) affected severely the retention characteristics. In most cases, the retention factors of the *ß*-methylated and non-methylated analogs are rather close, i.e., the retention properties are not influenced excessively by methylation at the *ß*-position.

The dependence of enantioselectivity on eluent composition is influenced by the structural characteristics of both the analyte and the selector; however, for the majority of analytes, no pronounced correlation with the eluent composition was observed (Figure S1). Regarding the enantioselectivities, it can be stated that the ZWIX(+) and ZWIX(−) columns have superior efficiency compared to the ZWIX(+A) and ZWIX(−A) columns. (The latter ones could provide only partial resolutions for some of the studied analytes.) The ZWIX(−) column provided the highest *α* and *R_S_* values for all analytes studied. Enantioselective interactions are significantly influenced by the relative configurations of the methyl and amino groups in the analytes, as indicated by the selectivity factors. Interestingly, the chiral recognition mechanisms of the ZWIX(+) and ZWIX(−) columns showed outstanding sensitivity to the relative configurations of the vicinal CH_3_ and NH_2_ groups. Enantiomer pairs with *anti* configurations, in all cases, were separated with selectivities much higher than those of their *syn* configuration counterparts. The selectivity values of the Tic enantiomer pairs were outstanding in comparison with others, suggesting that the ring closure through the methylene bridge in Tic restricts certain rotational conformations of the compound and fixes a more rigid structure. This restriction influences the enantioselective interactions through steric effects. Namely, the *anti* configuration ensures an exceptional fit to the *Cinchona* alkaloid-based selectors. It is important to note that aromatic interactions are not likely to play a major role in either nonselective or enantioselective interactions, as the chromatographic behavior of nonaromatic Cha enantiomer pairs is consistent with that of the other compounds. The enantioselectivities of the non-methylated analogs were consistently lower than those of the *ß*-methylated *anti* configuration analogs, yet higher than those observed for the *syn* configuration analogs.

As regards resolution, *R_S_* values, in most cases, followed the tendency observed for enantioselectivity, i.e., ZWIX(+) and ZWIX(−) columns had better resolution ability compared to the ZWIX(+A) and ZWIX(−A) columns, and enantiomer pairs with *anti* configurations were separated with higher resolution than those of their *syn* configuration counterparts (Figure S1). However, a special behavior was observed with respect to the change in the *R_S_* value with the change in the composition of the eluent: it reached its maximum value around the MeOH/MeCN (50:50, *v*/*v*) eluents containing FA (50 mM) and TEA (25 mM). Therefore, a comparison of the efficiency of different columns was carried out with this mobile phase composition (Figure 5B).

The elution order is of paramount importance in the separation of stereoisomers. The reliability of both qualitative identification and quantification can depend significantly on elution, namely, whether the minor impurity elutes before or after the major component. Three of the five chiral centers of quinine and quinidine have the same configuration, while two carbon atoms, which are crucial for chiral recognition, have opposite configurations, that is, the two molecules are in a pseudoenantiomeric relationship. In this case, however, they are diastereoisomers to each other [43,44]. Our results from the four zwitterionic columns demonstrated the pseudoenantiomeric character in all cases; a reversed enantiomer elution order (EEO) was recorded on both between the ZWIX(+) and ZWIX(−) columns and between the (ZWIX+A) and ZWIX(−A) columns. It is worth highlighting that an identical EEO was observed on the ZWIX(+) and ZWIX(+A) columns, as well as on the ZWIX(−) and ZWIX(−A) columns. This indicates that the change in the configuration of the cation exchanger ACHSA subunit has no major effect on the overall EEO; in other words, the absolute configuration of the *Cinchona* (quinine or quinidine) selector subunit plays a determining role in chiral recognition. It is also noteworthy that the *ß*-methylation of α-amino acid analytes has no significant effect on the EEO; the non-methylated analogs eluted with the same EEO as the methylated ones. It should also be pointed out that these observations are opposite to those published earlier [43]. In the case of cyclic *ß^3^*-amino acids, the configuration change in the ACHSA subunit site resulted in a reversed EEO, supporting a strong electrostatic interaction between the deprotonated ACHSA unit and the protonated amino group of the cyclic *ß^3^*-amino acids. Compared to cyclic amino acids, the ionizable groups of the analytes studied here are attached to an alkyl chain, allowing greater rotational freedom and easier adaptation to the relatively rigid structure of the ACHSA unit. Cyclic *ß*-amino acids, due to their more rigid backbone compared to acyclic α-amino acids, exhibit limited conformational flexibility, which enhances the sensitivity of their chiral recognition to steric and electronic modifications such as *ß*-methylation. This rigidity can lead to a reorganization of the dominant diastereomeric interactions upon methyl substitution, resulting in elution order reversal. In contrast, acyclic α-amino acids possess greater conformational freedom, allowing them to maintain similar interaction patterns with the chiral selector even after *ß*-methylation, thereby preserving their original elution order.

**Figure 6:**
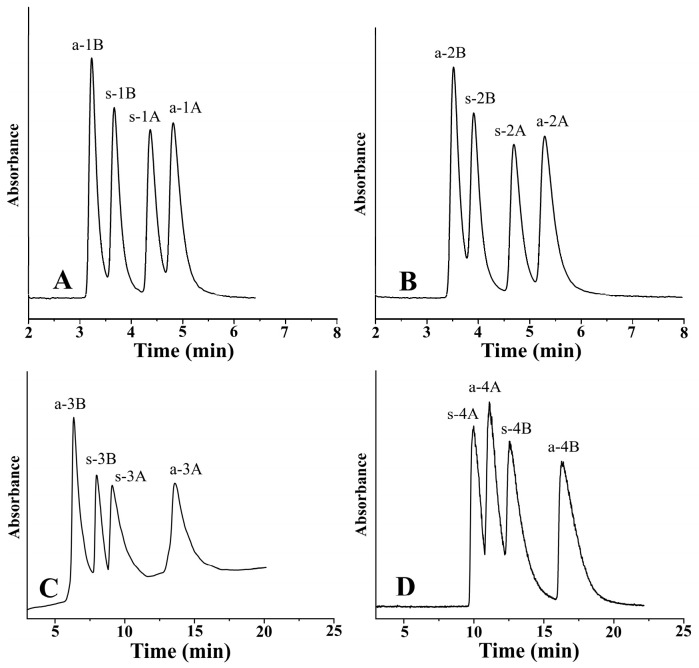
Representative chromatograms. Chromatographic conditions: (**A**) analyte, 1—*ß*-Me-Phe; column, Chiralpak ZWIX(−); mobile phase, MeOH/MeCN (60:40, *v*/*v*); additives, 50 mM FA + 25 mM TEA; flow rate, 0.6 mL min^−1^; detection, 254 nm; temperature, −5 °C. (**B**) Analyte, 2—*ß*-Me-Tyr; column, Chiralpak ZWIX(−); mobile phase, MeOH/MeCN (60:40, *v*/*v*); additives, 50 mM FA + 25 mM TEA; flow rate, 0.6 mL min^−1^; detection, 254 nm; temperature, −5 °C. (**C**) Analyte, 3—*ß*-Me-Tic; column, Chiralpak ZWIX(−); mobile phase, MeOH/MeCN (20:80, *v*/*v*); additives, 50 mM FA + 25 mM TEA; flow rate, 1.2 mL min^−1^; detection, 254 nm; temperature, 50 °C. (**D**) Analyte, 4—*ß*-Me-Cha; column, Chiralpak ZWIX(+); mobile phase, MeOH/MeCN (30:70, *v*/*v*); additives, 50 mM FA + 25 mM TEA; flow rate, 1.0 mL min^−1^; detection, CAD; temperature, −5 °C.

In the course of peptide synthesis, racemization frequently occurs. Due to this possible side effect, there is a great need to separate the stereoisomers in a single chromatographic run for analyzing the enantiomeric composition of amino acids in peptides after peptide hydrolysis. Figure 6 depicts the separation of four stereoisomers in a single chromatographic run under the optimized conditions.

### 2.5. Effect of Temperature on the Separation Performance

Temperature, which influences both the kinetic and thermodynamic aspects of the chromatographic process, may have significant impacts on retention and separation [45,46]. As the temperature increases, the viscosity of the mobile phase decreases, while the solute diffusion coefficient increases, leading to enhanced kinetic efficiency through faster mass transfer processes, a phenomenon known as the kinetic effect. On the other hand, temperature influences the partition coefficient of the solute, which determines how it distributes between the stationary and mobile phases, an effect commonly known as the thermodynamic effect. As the temperature increases, the interaction strength between the solute and the stationary phase generally weakens, leading to a reduction in the retention time. At higher temperatures, separations may become faster because of reduced retention times and improved kinetics. However, there can be trade-offs, such as reduced selectivity or resolution, particularly in chiral separations. Therefore, optimizing the temperature is crucial to balance separation efficiency, selectivity, and the analysis time. Importantly, studying this behavior can provide deeper insights into the retention mechanism.

The effects of temperature on retention and separation in chromatographic systems are typically described using the van’t Hoff equation,

(2) $$\ln\;\alpha =-\frac{\Delta \left(\Delta H^{0}\right)}{RT}+\frac{\Delta (\Delta S^{0})}{R}$$

where *α* is the selectivity factor, *Δ*(*ΔH*^0^) and *Δ*(*ΔS*^0^) are the differences in standard enthalpy and standard entropy, respectively, *R* is the universal gas constant, and *T* is the temperature in Kelvin. Using the plots of ln*α* vs. *T*^−1^ and calculating the values of *Δ*(*ΔH*^0^) and *Δ*(*ΔS*^0^) for pairs of enantiomers, uncertainties related to the determination of the phase ratio can be eliminated [47]. However, as highlighted in an excellent review by Asnin and Stepanova [48], this simplified approach suffers from several limitations. When all these limitations are considered, we believe that the evaluation of chromatographic characteristics obtained under identical conditions for model compounds with significant structural similarity is an applicable method that can yield valuable insights into the separation mechanism.

The influence of temperature on the chromatographic parameters of all analytes was investigated on ZWIX(+) and ZWIX(−) CSPs applying (i) MeOH and (ii) MeOH/MeCN (50:50, *v*/*v*) eluents containing FA (50 mM) and TEA (25 mM) in all cases. (The temperature dependent enantioslectivities are listed in Tables S2–S5). As mentioned above, by plotting ln*α* against *T^−1^*, *Δ*(*ΔH*^0^) and *Δ*(*ΔS*^0^) were determined in the temperature range of 5–50 °C, while *Δ*(*ΔG*^0^) was calculated for 25 °C on the basis of the values of *Δ*(*ΔH*^0^) and *Δ*(*ΔS*^0^). Confidence intervals were calculated to provide the statistical background for the data evaluation. When comparing the data listed in Table 1 and Table 2, several interesting conclusions can be drawn. All thermodynamic parameters were negative, with a single notable exception. Enantiomers of *ß*-MeTic with the *syn* configuration on the ZWIX(−) CSP applying MeOH/MeCN (50:50, *v*/*v*) eluent showed an unusual chromatographic behavior. That is, increased retention was observed with increasing temperature for both enantiomers, accompanied by increased enantioselectivity. In this case, the positive *Δ*(*ΔH^0^*) could be compensated by a rather high *Δ*(*ΔS*^0^), suggesting that the solvent molecules released from the CSP and the analyte, when the analyte is associated with the SO, may contribute significantly to the overall energetics. For all other analytes, enantioselectivity decreased (in a few cases, it did not change significantly) with increasing temperature and both *Δ*(*ΔH*^0^) and *Δ*(*ΔS*^0^) were negative.

It is worth noting that the most negative *Δ*(*ΔH*^0^) and *Δ*(*ΔG*^0^) values were determined for the enantiomers of *ß*-MeTic with the *syn* configuration, independently of the eluent composition and CSP configuration. This suggests that the adsorption of the enantiomers of *ß*-MeTic with the *syn* configuration is more favored compared to the other analytes studied.

Comparing the thermodynamic parameters of the enantiomers with *syn* and *anti* configurations, it is relevant to point out that more negative *Δ*(*ΔH*^0^) and *Δ*(*ΔG*^0^) values were obtained for the enantiomer pairs with the *anti* configuration in both eluent systems with both CSPs. This highlights an important conclusion: that is, steric interactions may outperform all other interactions.

When comparing the two zwitterionic CSPs, it can be stated that, in most cases, ZWIX(−) can be characterized with more negative *Δ*(*ΔH*^0^) and *Δ*(*ΔG*^0^) values than ZWIX(+) CSP, suggesting that in both eluent systems, ZWIX(−) can provide a better fit for the studied analytes.

Comprehensive studies investigating the impact of the mobile phase composition on the thermodynamic aspects of chiral separations are scarce in the scientific literature. Asnin et al. investigated the effect of the MeOH content on a Chirobiotic R column using MeOH/H_2_O-based eluents [49]. Their study revealed divergent correlations between the MeOH content and the thermodynamic parameters of the dipeptides examined. Comparing the thermodynamic parameters obtained for the two different eluent systems, more negative *Δ*(*ΔH*^0^) and *Δ*(*ΔS*^0^) values were obtained in MeOH (100%, *v*) in most cases, suggesting that enthalpy-driven separations are favored, independently of the eluent MeCN content. However, the values of *Δ*(*ΔG*^0^) were quite close, indicating that the changes in the enthalpy and entropy may compensate each other.

To assess the contributions of the enthalpy and entropy terms to adsorption, Q values [*Δ*(*ΔH°*)/[*T* × *Δ*(*ΔS*^0^)] at 298 K were also calculated. As illustrated in Table 3, in both eluents, higher Q values were determined for the ZWIX(−) CSP, showing preferred adsorption for the quinidine-based CSP. It is also interesting to highlight that markedly higher Q values were obtained when the eluent contained MeCN. Therefore, the contribution ratio of enthalpy to the whole enantioseparation is strongly affected by the MeCN content.

## 3. Materials and Methods

### 3.1. Chemicals and Reagents

Except for racemic and L-Phe, and racemic and L-Tyr, which were purchased from Sigma-Aldrich (St. Louis, MO, USA), all other analytes were prepared in our laboratory. (Preparation conditions and all other necessary data can be found in the Supporting Information file). HPLC-grade MeCN and MeOH, a methanolic solution of NH_3_, and analytical grade EA, DEA, TEA, formic acid (FA), and acetic acid (AcOH) were purchased from VWR International (Radnor, PA, USA). Ultrapure water was obtained from an Ultrapure Water System, Puranity TU UV/UF (VWR International, Radnor, PA, USA).

### 3.2. Apparatus and Chromatography

To perform HPLC measurements, two chromatographic systems were applied. The 1100 Series HPLC system from Agilent Technologies (Waldbronn, Germany) consisted of a solvent degasser, a pump, an autosampler, a column thermostat, a photodiode array UV-Vis detector, and a corona-charged aerosol detector (CAD) from ESA Biosciences, Inc. (Chelmsford, MA, USA). Data acquisition and analysis were performed with Agilent Technologies ChemStation chromatographic data software (version A. 09.03., Agilent Technologies Inc., Santa Clara, CA, USA). The second HPLC system was a Shimadzu Prominence HPLC system (Shimadzu Corporation, Kyoto, Japan) equipped with a CBM-20A system controller, a DGU-20A solvent degasser, an LC-20AB binary pump, an SPD-M20A photodiode array detector, a CTO-20AC column oven, and a SIL-20AC autosampler. The LabSolution chromatographic data software (version 5.111, Shimadzu Corporation, Kyoto, Japan) allowed the acquisition and processing of chromatographic data.

The Chiralpak ZWIX(+) and ZWIX(−) zwitterionic columns and the QN-AX, and QD-AX anion exchanger columns (150 × 3 mm I.D., 3 μm) were all from Chiral Technologies Europe (Illkirch, France). Details of the preparation of cation exchangers (DML-*RR* and DCL-*SS*, 150 × 4 mm I.D., 5 μm) and the zwitterionic ZWIX(+A) and ZWIX(−A) (150 × 3 mm I.D., 3 μm) were previously published [50,51,52]. The structures of the zwitterionic CSPs are depicted in Figure 2.

Stock solutions of amino acids (1 mg mL^−1^) were prepared by dissolving them in MeOH and further diluting them with the mobile phase. The hold-up times (*t*_0_) of the columns were determined by injecting acetone dissolved in MeOH at each investigated temperature and eluent composition. The flow rate was set at 0.6 mL min^−1^ with a column temperature of 25 °C, unless stated otherwise.

## 4. Conclusions

In this study, a systematic investigation was performed to evaluate both enantiorecognition and stereorecognition characteristics of *ß*-methyl-substituted unusual amino acids separated on ion exchanger-based chiral stationary phases. Both single-ion (cation and anion) and zwitterionic ion exchangers were investigated, where the zwitterionic CSPs were found to perform most effectively. Optimal performance was achieved with methanol and mixtures of methanol and acetonitrile eluents, along with acid and base additives, to control ionic interactions. The structural characteristics of protonated amines, applied as additives, were found to significantly affect retention and selectivity rather than their basicity. In experiments with varying counterion concentrations, neither selectivity nor resolution exhibited significant changes, while retention decreased with increasing counterion concentrations, a behavior indicative of ion exchange mechanisms. The effective charges calculated around 0.2 are evidence for the double ion-pairing retention mechanism.

In terms of enantioselectivity, the ZWIX(+) and ZWIX(−) columns demonstrated superior efficiency compared to the ZWIX(+A) and ZWIX(−A) columns. Of these, the ZWIX(−) column exhibited the highest *α* and *R_S_* values for all analytes examined. Enantioselective interactions of the unusual amino acids studied were found to be significantly influenced by the relative configurations of their *ß*-methyl and amino groups. The ZWIX(+) and ZWIX(−) columns displayed high sensitivity to these configurations, exhibiting greater selectivity for enantiomer pairs with *anti* configurations than for those with *syn* configurations. The more negative *Δ(ΔH*^0^*)* and *Δ(ΔG*^0^*)* values obtained for enantiomer pairs with *anti* configurations also support the outstanding importance of steric interactions. The thermodynamic study revealed enthalpy-driven enantioseparations in most cases and supported the superiority of the ZWIX(−) CSP in providing a better fit for the unusual amino acids studied. Due to their pseudoenantiomeric characteristics, the use of zwitterionic stationary phases allows the elution order to be easily altered by simply switching columns, which offers considerable advantages, in particular, when the quantification of a component present at low concentrations is required.

## Figures and Tables

**Table 1:** Thermodynamic parameters, *Δ(ΔH*^0^*)*, *Δ(ΔS*^0^*)*, and *Δ(ΔG*^0^*)*, of *ß*-methyl-substituted unusual amino acids obtained with MeOH (100%, *v*) containing 25 mM TEA and 50 mM FA with zwitterionic CSPs.

Analyte	*Δ(ΔH*^0^*)* (kJ mol^−1^)		*Δ(ΔS*^0^*)* (J mol^−1^ K^−1^)		*Δ(ΔG*^0^*)*_298K_ (kJ mol^−1^)	
	a	b	a	b	a	b
a-1	−1.59 ± 0.10	−2.26 ± 0.13	−2.74 ± 0.34	−2.57 ± 0.42	−0.77 ± 0.14	−1.49 ± 0.18
s-1	-	−1.14 ± 0.05	-	−1.45 ± 0.15	-	−0.71 ± 0.07
0-1	−1.00 ± 0.03	−1.99 ± 0.06	−1.74 ± 0.11	−3.51 ± 0.19	−0.49 ± 0.05	−0.95 ± 0.08
a-2	−1.50 ± 0.09	−2.09 ± 0.07	−2.65 ± 0.28	−2.60 ± 0.24	−0.71 ± 0.12	−1.32 ± 0.10
s-2	−0.86 ± 0.04	−0.98 ± 0.10	−2.34 ± 0.13	−0.99 ± 0.34	−0.17 ± 0.06	−0.68 ± 0.14
0-2	−1.20 ± 0.10	−2.06 ± 0.06	−2.62 ± 0.34	−3.81 ± 0.21	−0.42 ± 0.15	−0.93 ± 0.09
a-3	−2.72 ± 0.27	−2.67 ± 0.16	−2.11 ± 0.86	−1.23 ± 0.53	−2.09 ± 0.37	−2.30 ± 0.22
s-3	-	−1.74 ± 0.10	-	−4.19 ± 0.33	-	−0.49 ± 0.14
0-3	−1.26 ± 0.05	−1.38 ± 0.15	−1.57 ± 0.18	−0.29 ± 0.50	−0.79 ± 0.08	−1.29 ± 0.21
a-4	−1.56 ± 0.07	−2.02 ± 0.17	−3.19 ± 0.22	−2.47 ± 0.56	−0.61 ± 0.09	−1.28 ± 0.24
s-4	−1.31 ± 0.12	−1.68 ± 0.05	−2.38 ± 0.41	−1.65 ± 0.18	−0.60 ± 0.17	−1.18 ± 0.08
0-4	−1.37 ± 0.06	−2.29 ± 0.10	−2.57 ± 0.20	−3.97 ± 0.31	−0.61 ± 0.08	−1.10 ± 0.13

Chromatographic conditions: columns, (a) ZWIX(+) and (b) ZWIX(−); flow rate, 0.6 mL min^−1^; detection, 220 nm.

**Table 2:** Thermodynamic parameters, *Δ(ΔH*^0^*)*, *Δ(ΔS*^0^*)*, and *Δ(ΔG*^0^*)*, of *ß*-methyl-substituted unusual amino acids obtained with MeOH/MeCN (50:50, *v*/*v*) containing 25 mM TEA and 50 mM FA with zwitterionic CSPs.

Analyte	*Δ(ΔH*^0^*)* (kJ mol^−1^)		*Δ(ΔS*^0^*)* (J mol^−1^ K^−1^)		*Δ(ΔG*^0^*)*_298K_ (kJ mol^−1^)	
	a	b	a	b	a	b
a-1	−1.37 ± 0.08	−1.65 ± 0.10	−1.41 ± 0.28	−0.28 ± 0.32	−0.95 ± 0.12	−1.56 ± 0.14
s-1	-	-	-	-	-	-
0-1	−0.75 ± 0.13	−1.03 ± 0.03	−0.52 ± 0.45	−0.45 ± 0.10	−0.60 ± 0.19	−0.89 ± 0.04
a-2	−1.66 ± 0.18	−1.53 ± 0.16	−1.74 ± 0.58	−0.21 ± 0.53	−1.14 ± 0.25	−1.47 ± 0.23
s-2	−0.66 ± 0.05	-	−0.69 ± 0.17	-	−0.45 ± 0.07	-
0-2	−1.30 ± 0.21	−1.39 ± 0.14	−2.43 ± 0.71	−1.53 ± 0.48	−0.58 ± 0.30	−0.94 ± 0.20
a-3	−1.91 ± 0.12	−2.27 ± 0.16	−1.82 ± 0.40	−0.25 ± 0.55	−1.36 ± 0.17	−2.20 ± 0.23
s-3	-	1.22 ± 0.07	-	4.50 ± 0.22	-	−0.12 ± 0.09
0-3	−0.85 ± 0.09	-	−0.38 ± 0.30	-	−0.74 ± 0.13	-
a-4	−1.20 ± 0.13	−1.47 ± 0.09	−1.34 ± 0.43	−0.64 ± 0.30	−0.80 ± 0.18	−1.28 ± 0.13
s-4	−1.04 ± 0.09	−1.18 ± 0.13	−1.44 ± 0.31	−0.41 ± 0.44	−0.61 ± 0.13	−1.05 ± 0.19
0-4	−0.97 ± 0.17	−1.84 ± 0.12	−1.01 ± 0.57	−2.48 ± 0.41	−0.67 ± 0.24	−1.10 ± 0.17

Chromatographic conditions: columns, (a) ZWIX(+) and (b) ZWIX(−); flow rate, 0.6 mL min^−1^; detection, 220 nm.

**Table 3:** Enthalpy/entropy contribution ratio (Q) of *ß*-methyl-substituted unusual amino acids obtained with zwitterionic CSPs applying different mobile phase systems.

Analyte	Q [*Δ(ΔH^0^)*/*T* × *Δ(ΔS*^0^*)*_298K_]			
	MeOH (100%, *v)*		MeOH/MeCN (50:50, *v*/*v)*	
	a	b	a	b
a-1	2.0	3.0	3.3	19.5
s-1	-	2.6	-	-
0-1	1.9	1.9	4.8	7.7
a-2	1.9	2.7	3.2	24.9
s-2	1.2	3.3	3.2	-
0-2	1.5	1.8	1.8	3.1
a-3	4.3	7.3	3.5	31.0
s-3	-	1.4	-	0.9
0-3	2.7	16.0	7.5	-
a-4	1.6	2.7	3.0	7.7
s-4	1.8	3.4	2.4	9.6
0-4	1.9	1.9	3.2	2.5

Chromatographic conditions: columns, (a) ZWIX(+) and (b) ZWIX(−); flow rate, 0.6 mL min^−1^; detection, 220 nm.

## Data Availability

All data generated or analyzed during this study are included in the article/Supplementary Material.

## Supplementary Materials

The following supporting information can be downloaded at https://www.mdpi.com/article/10.3390/ijms26094004/s1. References [53,54,55,56,57] are cited in the supplementary materials.

## Abbreviations

The following abbreviations are used in this manuscript:

AcOH	Acetic acid
ACHSA	Aminocyclohexanesulfonic acid
CAD	Corona-charged aerosol detector
CSP	Chiral stationary phase
DEA	Diethyl amine
EA	Ethyl amine
EEO	Enantiomeric elution order
FA	Formic acid
HPLC	High-performance liquid chromatography
MeCN	Acetonitrile
PIM	Polar ionic mode
QD	Quinidin
QN	Quinin
TEA	Triethyl amine
