# Supplementary material for: High-Performance Liquid Chromatographic Separation of Stereoisomers of ß-Methyl-Substituted Unusual Amino Acids Utilizing Ion Exchangers Based on Cinchona Alkaloids
Source: Int J Mol Sci. 2025 Apr 23;26(9):4004. doi: 10.3390/ijms26094004 (PMC12072104; doi:10.3390/ijms26094004)
Supplement: Supplementary file 1 [file ijms-26-04004-s001.zip › ijms-3542766-supplementary.pdf]

Supporting Information for

High-performance liquid chromatographic separation of stereoisomers of *ß*-methyl-substituted unusual amino acids utilizing ion exchangers based on *Cinchona* alkaloids

Gábor Németi[1], Róbert Berkecz[1], Dániel Ozsvár[1], Zsolt Szakonyi[2], Wolfgang Lindner[3], Aleksandra Misicka[4], Dagmara Tymecka[4], Géza Tóth[5], Antal Péter[1], István Ilisz[1*]

[1]Institute of Pharmaceutical Analysis, University of Szeged, H-6720 Szeged, Somogyi u. 4, Hungary

[2]Institute of Pharmaceutical Chemistry, University of Szeged, H-6720 Szeged, Eötvös u. 6, Hungary

[3]Department of Analytical Chemistry, University of Vienna, Währinger Strasse 38, 1090 Vienna, Austria

[4]University of Warsaw, Faculty of Chemistry, Pasteura str. 1, 02-093 Warsaw, Poland

[5]Institute of Biochemistry, Biological Research Centre, H-6725 Szeged, Temesvári krt. 62, Hungary

Corresponding author: István Ilisz
Institute of Pharmaceutical Analysis, University of Szeged, Somogyi B. u. 4, H-6720 Szeged, Hungary
E-mail: ilisz.istvan@szte.hu

Table S1
Chromatographic data for the separation of stereoisomers of *β*-methyl-substituted unusual amino acids applying ZWIX(–) column with different acid additives

	k_1		α		R_S	
**Analyte**	**AcOH**	**FA**	**AcOH**	**FA**	**AcOH**	**FA**
**a-1**	1.91	2.10	1.77	1.79	2.06	2.77
**s-1**	2.82	3.08	1.00	1.00	0.00	0.00
**a-2**	2.35	2.41	1.76	1.76	1.64	2.67
**s-2**	2.59	2.65	1.34	1.35	0.99	1.57
**a-3**	1.76	1.97	2.32	2.34	3.15	4.32
**s-3**	1.91	2.16	1.07	1.09	0.23	0.39
**a-4**	2.62	2.66	1.62	1.63	2.60	3.30
**s-4**	2.51	2.62	1.49	1.49	1.63	2.24

Chromatographic conditions: column, ZWIX(–); mobile phase, MeOH/MeCN (50/50, *v/v*) containing 25 mM TEA and 50 mM AcOH or FA; flow rate, 0.6 mL min^{-1}; detection, 220 nm.

Table S2.
Effect of temperature on the enantioselectivity of the ZWIX(+) column in MeOH/MeCN (50/50 *v/v*) eluent

**Analyte**	**5 °C**	**10 °C**	**20 °C**	**30 °C**	**40 °C**	**50 °C**
**a-1**	1.52	1.51	1.48	1.45	1.43	1.40
**s-1**	1.12	1.10	1.10	1.10	1.13	1.17
**0-1**	1.29	1.28	1.28	1.27	1.26	1.25
**a-2**	1.67	1.61	1.60	1.57	1.54	1.51
**s-2**	1.22	1.22	1.21	1.20	1.19	1.18
**0-2**	1.31	1.31	1.28	1.24	1.23	1.22
**a-3**	1.80	1.78	1.75	1.71	1.67	1.63
**s-3**	1.05	1.05	1.04	1.03	1.02	1.00
**0-3**	1.37	1.36	1.35	1.34	1.33	1.31
**a-4**	1.31	1.42	1.39	1.38	1.35	1.33
**s-4**	1.31	1.31	1.29	1.27	1.25	1.24
**0-4**	1.33	1.33	1.32	1.32	1.28	1.27

Chromatographic conditions: column, ZWIX(+); mobile phase, MeOH/MeCN (50/50 *v/v*) containing 25 mM TEA and 50 mM FA; flow rate, 0.6 mL min^{-1}; detection, 220 nm, CAD; temperature, 5-50 °C.

Table S3.
Effect of temperature on the enantioselectivity of the ZWIX(+) column in MeOH (100%, *v*) eluent

Analyte	5 °C	10 °C	20 °C	30 °C	40 °C	50 °C
**a-1**	1.42	1.41	1.37	1.35	1.32	1.30
**s-1**	1.05	1.00	1.00	1.00	1.00	1.00
**0-1**	1.25	1.24	1.23	1.21	1.19	1.18
**a-2**	1.39	1.38	1.34	1.32	1.29	1.27
**s-2**	1.10	1.09	1.07	1.06	1.05	1.04
**0-2**	1.23	1.21	1.19	1.17	1.16	1.14
**a-3**	1.76	1.74	2.32	2.28	2.21	2.13
**s-3**	1.09	1.06	1.00	1.00	1.00	1.00
**0-3**	1.43	1.41	1.39	1.36	1.34	1.32
**a-4**	1.34	1.32	1.28	1.26	1.24	1.21
**s-4**	1.32	1.31	1.28	1.26	1.24	1.22
**0-4**	1.33	1.31	1.29	1.27	1.24	1.22

Chromatographic conditions: column, ZWIX(+); mobile phase, MeOH/MeCN (100/0 *v/v*) containing 25 mM TEA and 50 mM FA; flow rate, 0.6 mL min^{-1}; detection, 220 nm, CAD; temperature, 5-50 °C.

Table S4.
Effect of temperature on the enantioselectivity of the ZWIX(–) column in MeOH/MeCN (50/50 *v/v*) eluent

Analyte	5 °C	10 °C	20 °C	30 °C	40 °C	50 °C
**a-1**	1.96	1.94	1.90	1.86	1.82	1.78
**s-1**	1.35.	1.35	1.35	1.36	1.35	1.34
**0-1**	1.48	1.46	1.45	1.42	1.41	1.39
**a-2**	1.86	1.87	1.82	1.80	1.76	1.72
**s-2**	1.36	1.39	1.37	1.38	1.40	1.35
**0-2**	1.52	1.49	1.49	1.45	1.41	1.40
**a-3**	2.43	2.49	2.45	2.39	2.33	2.27
**s-3**	1.00	1.02	1.04	1.06	1.08	1.09
**0-3**	1.65	1.66	1.69	1.67	1.66	1.64
**a-4**	1.75	1.72	1.69	1.66	1.62	1.50
**s-4**	1.56	1.55	1.54	1.53	1.50	1.48
**0-4**	1.63	1.62	1.58	1.54	1.51	1.47

Chromatographic conditions: column, ZWIX(–); mobile phase, MeOH/MeCN (50/50 *v/v*) containing 25 mM TEA and 50 mM FA; flow rate, 0.6 mL min^{-1}; detection, 220 nm, CAD; temperature, 5-50 °C.

Table S5.
Effect of temperature on the enantioselectivity of the ZWIX(–) column in MeOH (100%, *v*) eluent

Analyte	5 °C	10 °C	20 °C	30 °C	40 °C	50 °C
**a-1**	1.93	1.92	1.87	1.80	1.75	1.70
**s-1**	1.37	1.36	1.35	1.32	1.30	1.28
**0-1**	1.58	1.53	1.49	1.44	1.41	1.38
**a-2**	1.81	1.79	1.73	1.67	1.63	1.60
**s-2**	1.36	1.34	1.34	1.31	1.29	1.28
**0-2**	1.57	1.52	1.48	1.43	1.40	1.37
**a-3**	2.74	2.74	2.53	2.48	2.40	2.33
**s-3**	1.28	1.26	1.23	1.20	1.18	1.16
**0-3**	1.77	1.74	1.70	1.67	1.64	1.62
**a-4**	1.75	1.73	1.70	1.66	1.62	1.57
**s-4**	1.70	1.67	1.63	1.59	1.56	1.53
**0-4**	1.69	1.64	1.57	1.54	1.49	1.45

Chromatographic conditions: column, ZWIX(–); mobile phase, MeOH/MeCN (100/0 *v/v*) containing 25 mM TEA and 50 mM FA; flow rate, 0.6 mL min^{-1}; detection, 220 nm, CAD; temperature, 5-50 °C.

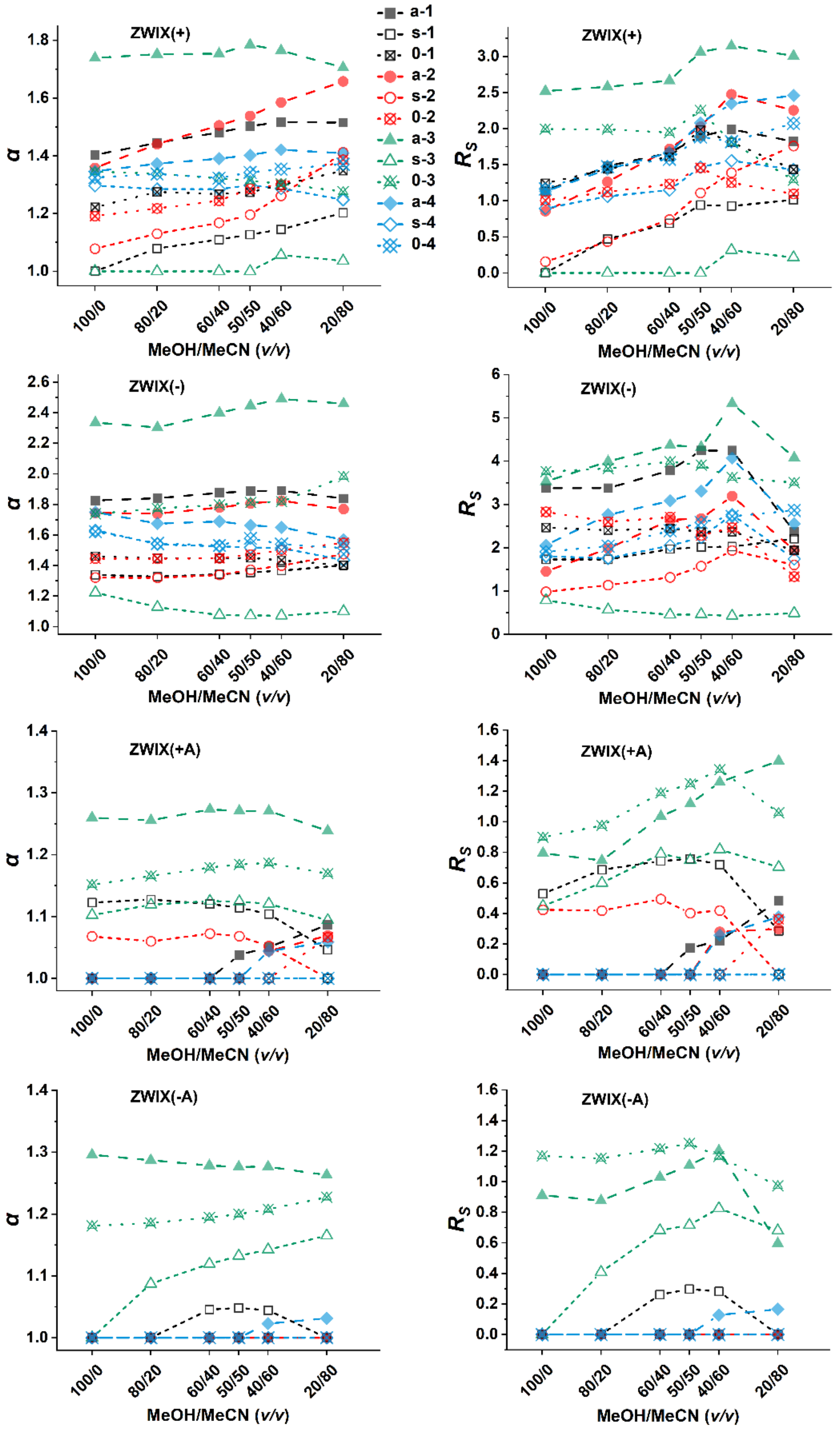

Figure S1

Effects of the mobile phase composition on the selectivity and resolution

Chromatographic conditions: columns, Chiralpak ZWIX(+), ZWIX(–), ZWIX(+A), ZWIX(–A); mobile phase, MeOH/MeCN (100:0 – 20:80, *v/v*); additives, 50 mM FA + 25 mM TEA; flow rate, 0.6 mL min^{-1}; detection, 254 nm, and CAD; temperature, 25 °C; analyte, 1: *β*-Me-Phe, 2: *β*-Me-Tyr, 3: *β*-Me-Tic, 4: *β*-Me-Cha;

Experimentals
NMR data and preparation conditions for the studied analytes

β-Me-Phe (1)

References:
Csaba Tömböly, Katalin E. Kövér, Antal Péter, Dirk Tourwé,| Dauren Biyashev, Sándor Benyhe, Anna Borsodi, Mahmoud Al-Khrasani, András Z. Rónai, and Géza Tóth
Structure-activity study on the Phe side chain arrangement of endomorphins using conformationally constrained analogues
Journal of Medical Chemistry 2004 (47) 735-743; DOI: 10.1021/jm0310028

a-1: (DMSO-d_6) δ 7.66 (NH, J = 8.6 Hz), 7.1-7.3 (aromatic), 4.49 (H^{α}, J = 9.0 Hz), 3.13 (H^{β}, J = 7.2 Hz), 1.20 (CH_3).

s-1: (DMSO-d_6) δ 7.78 (NH, J = 8.9 Hz), 7.1-7.3 (aromatic), 4.52 (H^{α}, J = 9.2 Hz), 3.09 (H^{β}, J = 7.2 Hz), 1.19 ($H^{\gamma,\gamma'}$).

Optically pure peptides were obtained by semipreparative RP-HPLC. The absolute configuration of β-MePhe in the peptides was determined by chiral TLC analyses of the acidic hydrolysates of peptides. The (2S)-β-MePhe isomers had higher R_f values than the corresponding (2R)-isomers in an eluent mixture of acetonitrile-methanol-water (4:1:1). Comparison of the TLC data with the RP-HPLC chromatograms clearly revealed that the compound eluting first from the reversed-phase column contained (2S)-β-MePhe isomer. The pure erythro-(2S,3S and 2R,3R)- and the pure threo-(2S,3R and 2R,3S)-β-MePhe racemates were obtained by fractional crystallization of the isomeric mixture prepared by the method of Kataoka.

β-Me-Tyr (2) and **β-Me-Tic (3)**

References:
Dirk Tourwé, Els Mannekens, Trang Nguyen Thi Diem, Patricia Verheyden, Hendrika Jaspers, Géza Tóth, Antal Péter,| István Kertész, Gabriella Török,| Nga N. Chung, and Peter W. Schiller
Side chain methyl substitution in the δ-opioid receptor antagonist TIPP has an important effect on the activity profile
Journal of Medical Chemistry 1998 (41) 5167-5176; DOI: 10.1021/jm981011u

a-2: (D_2O/TFA) δ 13.6 (s, 1H, COOH), 6.75 (d, 2H, aromatic, J = 8.6 Hz), 6.43 (d, 2H, aromatic, J = 8.6 Hz), 3.65 (d, 1H, H^{α}, J = 7.2 Hz), 3.07 (m, 1H, H^{β}), 0.94 (d, 3H, CH_3, J = 7.2 Hz).
s-2: (D_2O/TFA) δ 13.6 (s, 1H, COOH), 6.81 (d, 2H, aromatic, J = 8.6 Hz), 6.43 (d, 2H, aromatic, J = 8.6 Hz), 4.11 (d, 1H, H^{α}, J = 7.2 Hz), 3.37 (m, 1H, H^{β}), 1.31 (d, 3H, CH_3, J = 7.0 Hz).
a-3: (D_2O) δ 7.57-7.5 (m, 4H, aromatic), 4.53 (d, 2H, H_1, J = 18 Hz), 3.89 (m, 1H, H_3), 3.54 (m, 1H, H_4), 1.59 (d, 3H, CH_3, J = 7.5 Hz).
s-3: (D_2O) δ 7.52-7.35 (m, 4H, aromatic), 4.55 (d, 2H, H_1, J = 18 Hz), 4.47 (m, 1H, H_3), 3.74 (m, 1H, H_4), 1.39 (d, 3H, CH_3, J = 7.5 Hz).

Identification of enantiomers of β-methyl amino acids in peptides involved three steps: acid hydrolysis of peptides, derivatization of the resulting amino acids with chiral derivatizing reagent GITC or FDAA, and separation and identification of derivatized amino acids by HPLC. The separation of derivatized amino acids was carried out on a Vydac 218TP54 C_{18} column.

The peaks of derivatized *β*-methyl amino acids were identified by application of standards of (2*R*,3*S*)- and (2*R*,3*R*)-*β*-methyl amino acids, obtained by L-AA oxidase digestion of the racemic erythro or threo isomers.

<u>*β*-Me-Cha (4)</u>

<u>References:</u>

Géza Tóth, Enikő_Ioja, Csaba Tömböly, Steven Ballet, Dirk Tourwé, Antal Péter, Tamás Martinek,| Nga N. Chung, Peter W. Schiller, Sándor Benyhe, and Anna Borsodi
β-Methyl substitution of cyclohexylalanine in Dmt-Tic-Cha-Phe peptides results in highly potent δ opioid antagonists
Journal of Medical Chemistry 2007 (50) 328-333; DOI: 10.1021/jm060721u

a-4: ^{1}H-NMR (DCl/DMSO-*d*$_6$) δ 8.60 (m, NH_3^+), 3.82 (d, 1H, H^α, *J* = 4.0 Hz), 1.79-0.78 (m, 12H, H^β, cyclohexyl-CH), 0.85 (d, 3H, CH_3, *J* = 7.0 Hz). ^{13}C NMR (DCl/DMSO-*d*$_6$) δ 170.2 (COOH), 54.2 (C^α), 39.7 (C^β), 38.4 (cyclohexyl-CH), [31.0, 29.2, 26.2, 26.1, 25.9] (cyclohexyl-CH_2), 12.0 (CH_3).
s-4: ^{1}H-NMR (DCl/DMSO-*d*$_6$) δ 8.42 (m, NH_3^+), 3.82 (d, 1H, H^α, *J* = 4.0 Hz), 1.79 (m, 1H, H^β), 1.63-0.77 (m, 10H, cyclohexyl-CH_2), 1.32 (m, 1H, cyclohexyl-CH), 0.82 (d, 3H, CH_3, *J* = 7.0 Hz).
^{13}C NMR (DCl/DMSO-*d*$_6$) δ 171.2 (COOH), 54.5 (C^α), 39.3 (C^β), 37.6 (cyclohexyl-CH), [31.1, 29.3, 26.1(2C), 25.9] (cyclohexyl-CH_2), 12.0 (CH_3).

The racemic erythro-(2*S*,3*S* and 2*R*,3*R*) and threo-(2*S*,3*R* and 2*R*,3*S*)-*β*-MeCha diastereoisomers were obtained from the corresponding erythro- or threo-*β*-MePhe by hydrogenation at 60 psi H_2 and 50 °C using a PtO_2 catalyst in aqueous acetic acid solution, as was previously reported for the preparation of cyclohexylalanine from phenylalanine. The configuration of the *R*-carbon was determined directly by enzymatic digestion of the *β*-MeCha isomers with L-amino acid oxidase, followed by RP-HPLC separation of the chiral derivatized *β*-MeCha isomers or by chiral TLC separation of the amino acid isomers. The GITC derivatization was more useful, as it made it possible to separate all four *β*-MeCha isomers and, thus, the 2*R* and 2*S* diastereomers were unambiguously identified.

<u>Cha:</u>

To a solution of 0.50 g (3.03 mmol) of L-phenylalanine in a mixture of 5 mL of glacial acetic acid and 4 mL of water, 0.15 g of platinum oxide was added and the mixture was hydrogenated at 50 psi of hydrogen at 50 °C for 18 h by stirring in a Parr autoclave apparatus. The reaction mixture was cooled to room temperature and the semisolid mass dissolved by adding a 1:1 mixture of acetic acid and methanol (5 mL). The solution was filtered through a small pad of Celite and the pad was washed with additional methanol (5 mL). The combined filtrates were evaporated in vacuo and the remaining solid was triturated with anhydrous diethylether (2 x 5 mL) and filtered. (*S*)-2-Amino-3-hexahydrophenylpropionic acid was isolated as white crystalls: 0.42 g (81%), $[\alpha]^{25}_D$ = +9.8 (c 0.35, 1.0 M HCl), mp 213–217 °C, ^{1}H-NMR (D_2O; 500 MHz) 0.94-1.08 (m, 2H), 1.14-1.36 (m, 3H), 1.42-1.54 (m, 1H), 1.62-1.94 (m, 7H), 4.10 (dd, 6.4, 7.9 Hz, 1H).

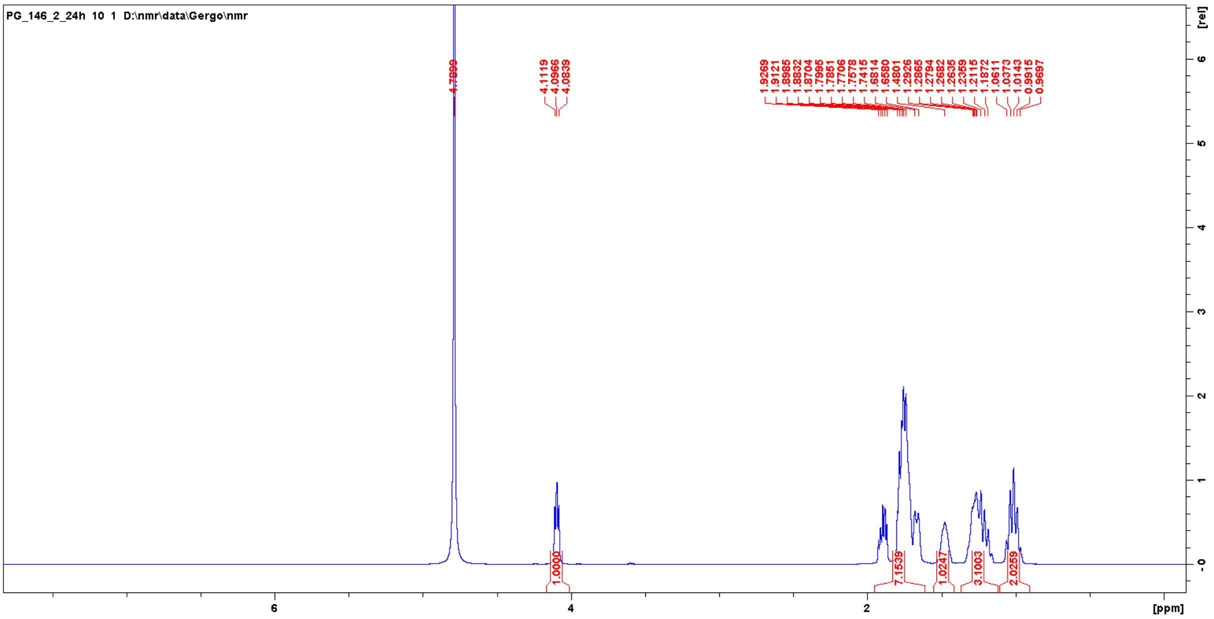

References:

1) Paul Francis Schuda, William J. Greenlee, P. K. Chakravarty, and Philip Eskola
A short and efficient synthesis of (3*S*,4*S*)-4-[(tert-Butyloxycarbonyl)amino]-5-cyclohexyl-3-hydroxypentanoic acid ethyl ester
Journal of Organic Chemistry 1988 (53) 873-875; doi:10.1021/jo00239a036
2) Limin Shi, Chuangan Tao, Qin Yang, Yong Ethan Liu, Jing Chen, Jianfeng Chen, Jiaxin Tian, Feng Liu, Bo Li, Yongling Du, and Baoguo Zhao
Chiral pyridoxal-catalyzed asymmetric biomimetic transamination of α-keto acids
Organic Letters 2015 (17) 5784-5787 doi: 10.1021/acs.orglett.5b02895
